# Differences in Stress and Anxiety Among Women With and Without Children in the Household During the Early Months of the COVID-19 Pandemic

**DOI:** 10.3389/fpubh.2021.688462

**Published:** 2021-09-01

**Authors:** Ally R. Avery, Siny Tsang, Edmund Y. W. Seto, Glen E. Duncan

**Affiliations:** ^1^Department of Nutrition and Exercise Physiology, Washington State University, Spokane, WA, United States; ^2^Department of Environmental and Occupational Health Sciences, University of Washington, Seattle, WA, United States

**Keywords:** COVID-19, perceived stress, anxiety, twins, mothers

## Abstract

The COVID-19 pandemic and resulting lockdowns have had a disproportionate impact on parents of children under 18, particularly women. Mandatory school closures and loss of childcare resulted in parents balancing work, teaching, and childcare needs. A number of studies have examined changes in mental health of parents, but to date no studies have compared the differences in stress and anxiety levels between women with and without children in the United States. Adult women from the Washington State Twin Registry (WSTR) (*N* = 1,014, pair *N* = 529) and mothers of twin children enrolled in the WSTR (*N* = 147) completed an online survey examining several health-related behaviors and outcomes and their self-reported changes due to COVID-19. We conducted two studies to examine the impact of children on stress and anxiety levels among women. In study 1, we assessed whether women living in households with children under the age of 18 have higher levels of stress and anxiety than those without children in their household. We found that perceived stress levels did not differ between women with and without children in the household, but anxiety levels were higher among women living with children than those without. In study 2, we assessed whether the correlation between children in the household and stress/anxiety is accounted for by non-random genetic and environmental selection effects, causal processes, or both using a sample of adult female twins. We found that the presence of children in the household was associated with higher levels of stress and anxiety. However, this association is confounded by genetic and shared environmental factors. Our findings highlight the need to provide supporting resources to women living with children in the household during and after the COVID-19 pandemic.

## Introduction

The coronavirus (COVID-19) has impacted nearly every country around the world since being declared a pandemic on March 11, 2020 (1). Parents and/or guardians with children under 18 years old in the household are among some of those most impacted by the mitigating strategies aimed to slow down the spread of the virus. In the US, 42 states ordered mandatory closures of all K-12 public schools for the 2019–2020 school year, with the remaining states either recommending closure or varying closures by school district (2). Although many schools made efforts to supplement the lack of formal classroom education with take-home packets/assignments, instructional materials on the internet, and/or online classrooms, disparities in access meant some households received better support for remote learning than others (3). Parents with children who struggled with distance learning experienced higher levels of anxiety and depression than parents whose children coped well with remote learning (4). Parents of young children have faced reduced availability in childcare services due to the temporary closure of childcare providing services and/or reduced contact with non-household members who may otherwise be offering childcare assistance (5–7), resulting in worsening mental health (8). Parents, especially women, working from home have reported struggling to balance working remotely with providing childcare, as well as monitoring children involved with at-home learning (9, 10). Overall, parental burnout has been reported during the COVID-19 pandemic as parents experienced an increase in demands with a decrease in available resources (11).

Research regarding the impact of parenting on mental health has shown inconsistent findings. In general, parents have reported higher levels of distress than those without children, with mothers reporting higher levels of distress and depressive symptoms than fathers (12–14). Arranging childcare is a major stressor for parents that has negative impacts on mental health, especially for working mothers (14–16). Other studies have found that parenthood is associated with improved mental health outcomes. For example, motherhood was associated with better mental health among a sample of Australian mothers between the age of 30 and 34 (17). Mothers with access to a support network (e.g., partner, family members, or other mothers) reported decreased levels of anxiety and stress (18). Nomaguchi and Milkie (19) showed that the benefits of parenthood were dependent on one's social integration (time spent with others), marital status, and gender. Levels of distress were found to be influenced by the quality of the parent-child relationship; parents who reported a close relationship with their child were also more likely to have enhanced well-being (20, 21). On the other hand, it has also been reported that parenthood was not associated with enhanced emotional well-being, with parents and non-parents reporting similar levels of depression (22).

Traumatic events and/or natural disasters can lead to an increase in demands on parents who may become less available for their children (23). These events may also impact their ability to interact with their children in a positive and consistent manner (24). After a massive flood in St. Louis, Missouri in the winter of 1982, parents reported higher levels of stress than their non-parent counterparts (25). Among a sample of parents living in the US, Mexico, and Canada who spent time in quarantine or isolation during the H1N1 pandemic in the spring of 2009, more than half of the participants were at risk of developing post-traumatic stress disorder (PTSD) or met the diagnostic criteria for PTSD (26).

Recent studies suggest that parents' mental health has suffered during the COVID-19 pandemic. In Italy, parents who found it difficult to accommodate their children's education during school closures were more stressed than those who did not (27), and women with children had higher levels of anxiety compared to women without children (28). A sample of pregnant and postpartum women, primarily residing in Canada, reported an increase in self-reported anxiety and depression about a month after COVID-19 was declared a pandemic (29). This finding was replicated among another sample of women residing primarily in Canada, with mothers of younger children reporting higher levels of anxiety than mothers of older children (30). A study of UK adults found that having children in the household was associated with higher levels of anxiety (31). COVID-19-related stressors, such as the parent's relationship with their partner and their child(ren)'s academics, were associated with an increase in perceived stress among US parents of children under 18 (32). Another study of adults residing in the US reported a small effect of number of children in the household on depression during the first few months of the COVID-19 pandemic; self-reported depression levels were slightly higher among those living with more children than those with fewer children in the household (33).

To date, no studies have examined levels of stress and anxiety during COVID-19 between women with and without children primarily residing in the US. Additionally, it remains unclear whether the association between children in the household and levels of stress and anxiety is attributable to non-random selection, causal effect, or both. The objective of the current study is 2-fold. First, we aimed to assess whether adult women living in households with children under the age of 18 have higher levels of stress and anxiety than those without children in the household. Second, we addressed whether the correlation between children in the household and women's stress/anxiety mediated by genetic and environmental confounds shared within twin pairs raised together. We limited our sample to women, because women have taken on most of the responsibility for household duties such as childcare, distance learning support, and housework during the COVID-19 pandemic (10). Our findings contribute to a growing body of literature showing the impact of the COVID-19 lockdown on mental health, specifically in women. We describe each study in detail below.

## Study 1

In study 1, we examined whether having children under the age of 18 residing in the household is associated with stress and anxiety levels among women during the COVID-19 pandemic. Use of “living with children under 18” has been used elsewhere to identify those who are actively parenting (34). We hypothesized that women with children in the household would have higher levels of stress and anxiety levels, compared to those living without children (31, 35). We further hypothesized that stress and anxiety levels would increase with the number of children in the household, such that women living with more children would be more stressed and anxious than those living with no or fewer children (31, 36).

### Methods

#### Participants

This study utilized data from two research samples among 1,161 adult women in the Washington State Twin Registry (WSTR). The WSTR is a community-based registry of twin pairs primarily recruited through Washington State Department of Licensing (DOL) records. The WSTR enrolls twin pairs across the lifespan. Adult twins over the age of 18 enroll themselves and youth twins under 18 are enrolled with their parent or guardian. Details about the WSTR's recruitment procedures and additional information are reported elsewhere (37–39).

Sample 1 consisted of 1,014 adult singleton (i.e., only one member of the twin pair completed the survey) women^1^ from the WSTR who completed an online survey examining several health-related behaviors and outcomes during the first few weeks after COVID-19 was declared a pandemic. The survey was sent to 12,173 adult individuals registered and active in the WSTR between March 26 and April 5, 2020. The individual response rate was 32.8%. Eight participants were missing responses to the question asking the number of children in the household and were subsequently excluded from this study. Sample 2 consisted of 147 mothers of twins ages 13 and younger who completed an online survey assessing health-related behaviors and outcomes for themselves and their children during the COVID-19 pandemic. The survey was sent to 475 parents/guardians registered in the WSTR between May 7 to May 24, 2020; the response rate was 33.1%. In summary, the analytic sample for study 1 consists of 1,006 adult women unrelated to each other; sample 1 includes women with and without children in their household, whereas sample 2 includes women with at least one pair of twins in their household. We combined data from the two samples to increase sample size.

#### Procedures

Invitations to participate in both online studies were sent via email to individuals registered in the WSTR. The invitation email included information about the study, and a link for them to complete the survey online. Participation was voluntary and no incentive was offered. Both studies were approved by the IRB at Washington State University. A wavier of documentation of consent was obtained, and consent was assumed by completing the questionnaire.

#### Measures

##### Number of children in the household

Children in the household was assessed with the question, “Currently, how many children (under the age of 18) live in your household?” Possible responses ranged from 0 to 10 or more children. The last option, 10 or more, was top-coded as 10 in this study.

##### Perceived Stress

The 10-item Perceived Stress Scale (PSS) (40) was used to assess perceived stress levels. Participants were asked about the frequency of a number of feelings and thoughts in the last 2 weeks, rating them on a 5-point Likert-type scale (0 = Never; 1 = Almost never; 2 = Sometimes; 3 = Fairly often; 4 = Very often). A total PSS score (range = 0–40) was obtained by summing across all scale items, with higher scores indicating higher levels of perceived stress. Cronbach's alpha for the PSS is 0.89 (95% CI: 0.88, 0.90) in the current study, suggesting good reliability.

##### Anxiety

Anxiety was assessed using the six-item anxiety subscale in the Brief Symptom Inventory (BSI) (41). Participants were asked to indicate how much discomfort each problem has caused them during the past 2 weeks including today on a 5-point Likert-type scale (0 = Not at all; 1 = A little bit; 2 = Moderately; 3 = Quite a bit; 4 = Extremely). A total anxiety score (range = 0 to 24) was computed by summing across all items, with higher scores reflect higher levels of anxiety. Internal consistency of the anxiety subscale was good in our study (Cronbach's alpha = 0.86, 95% CI: 0.85, 0.87).

##### Covariates

Participants' age, race, and number of adults in the household were included as covariates in the statistical analyses. Age referred to individuals' age at which they completed the survey; it was computed using the reported date of birth. Sex was self-reported as male or female. Race was coded as White or non-White based on participants' self-report on six response categories. The number of individuals in the household was assessed using the question, “Currently, how many adults (over the age of 18) including yourself live in your household?” Possible responses ranged from 1 to 10 or more. The last option, 10 or more, was top-coded as 10 in the current analyses.

#### Statistical Analysis

We reported the differences among the three groups of participants (sample 1 without children, sample 1 with children, and sample 2 with children). Differences among the three groups were examined using linear regression models (for continuous variables) and chi-square tests (for categorical variables).

We used a series of multiple regression models to investigate whether the presence of children in the household was associated with perceived stress and anxiety, with each outcome in separate models. First, we examined whether stress and anxiety levels were higher with the presence of children in the household. The independent variable of interest, children in the household, was modeled as a dichotomous variable (yes/no) in this set of regression models. Participants' age, race, and number of adults in the household were included as covariates. Considering the differences in ages across participants with and without children in the household, the interaction between children in the household (yes/no) and age was also included in the models.

Next, the models were re-estimated by modeling the number of children in the household as a continuous variable (range = 0–10), instead of a dichotomous variable. This next set of models allowed us to explore whether perceived stress and anxiety levels differed by the number of children in the household. Number of children in the household and age were centered to prevent collinearity issues.

In all the above models, perceived stress and anxiety were square root transformed due to skewness, and age was divided by 10 to allow variables to be on similar scales. All statistical analyses were performed in R 4.0.2 (42). The alpha level for testing hypotheses was set to 0.05.

### Results

#### Descriptive Statistics

Table 1 shows the descriptive statistics of select demographic characteristics of the women in this study, stratified by sample and presence of children in the household. On average, women in sample 2 had more children in the household (*M* = 2.8, *SD* = 1.0) than the women in sample 1 with children (*M* = 1.8, *SD* = 0.8; *p* < 0.001). This is expected, as women in sample 2 were parents and/or guardians of at least one pair of twin children (as described in the Methods section). Participants in sample 1 with no children in the household were, on average, older (*M* = 54.0, *SD* = 16.5) than individuals in sample 1 with children (*M* = 41.5, *SD* = 8.1) and respondents in sample 2 (*M* = 40.4, *SD* = 5.1; *p* < 0.001). The three groups of participants also differed in the number of adults in the household (*p* = 0.001), average perceived stress (*p* < 0.001) and anxiety (*p* = 0.026) levels.

**Table 1:** Descriptive statistics of select demographic characteristics, number of children and adults in household, perceived stress, and anxiety in two samples of women.

	**Sample 1**		**Sample 2**	
	**No kids**	**With kids**	**With kids**	
	***n* = 679**	***n* = 327**	***n =* 147**	***p***
Age	54.0 (*16.5*)	41.5 (*8.13*)	40.4 (*5.1*)	<0.001
	Range = 20.8–90	Range = 21.1–81.4	Range = 28.0–58.2	
White	650 (95.7%)	304 (93.0%)	140 (95.2%)	0.173
Number of children in household	0	1.8 (0.8)	2.8 (1.0)	<0.001
		Range = 1–6	Range = 2–8	
Number of adults in household	2.0 (*0.9*)	2.2 (*0.8*)	2.1 (*0.5*)	0.001
	Range = 1–8	Range = 1–6	Range = 1–5	
Perceived stress	11.6 (*7.1*)	13.1 (*6.9*)	14.4 (*6.8*)	<0.001
Anxiety	3.4 (*3.6*)	3.7 (*3.9*)	4.1 (*3.9*)	0.026

*Sample 1 consists of adult singleton twin respondents from the Washington State Twin Registry (WSTR). Sample 2 consists of mothers of twins enrolled in the WSTR. Means (standard deviations) are presented for continuous variables. Medians [ranges] are presented for the number of people in household. Frequencies (proportions) are presented for categorical variables*.

#### Presence of Children and Stress and Anxiety

As shown in Table 2, there was no main effect of children in the household on perceived stress (*b* = −0.57, *SE* = 0.31, *p* = 0.066), after controlling for age, race, number of adults in the household, and the interaction between children in the household and age. This means that perceived stress levels did not differ between women with and without children in the household. Age was negatively associated with stress (*b* = −0.29, *SE* = 0.02, *p* < 0.001), suggesting that younger women were, on average, more stressed than older women. There was no significant interaction between children in the household and age on perceived stress (*b* = 0.13, *SE* = 0.07, *p* = 0.072).

**Table 2:** Multiple regression models estimating the extent to which having children in the household is associated with perceived stress and anxiety.

	**Perceived stress**			**Anxiety**		
	***Est***	***SE***	***p***	***Est***	***SE***	***p***
Intercept	4.53	0.22	<0.001	2.63	0.20	<0.001
Children in household (yes)	−0.57	0.31	0.066	−0.75	0.29	0.011
Age	−0.29	0.02	<0.001	−0.26	0.02	<0.001
Race (White)	0.10	0.14	0.472	0.17	0.14	0.206
Number of adults in household	0.06	0.04	0.108	0.05	0.04	0.153
Children × Age	0.13	0.07	0.072	0.14	0.07	0.039
*R^2^*	0.133			0.109		

*Perceived stress and anxiety were square root transformed. Age was divided by 10. Age, race, and number of adults in household were included as covariates. R^2^, proportion of variance explained*.

We found a significant main effect of children in the household on anxiety (*b* = −0.75, *SE* = 0.29, *p* = 0.011), and a significant main effect of age on anxiety (*b* = −0.26, *SE* = 0.02, *p* < 0.001). There was also a significant interaction between children in the household and age on anxiety (*b* = 0.14, *SE* = 0.07, *p* = 0.039). Results suggested that the difference in anxiety levels between women with and without children in the household differs across women of different ages. Among women with no children, anxiety levels decreased with increasing age, meaning that younger women with no children were more anxious than older women with no children in the household. Anxiety levels also decreased with increasing age among women with children, however, the effect was much smaller.

We next explored whether stress and anxiety levels differ by the number of children in the household (Table 3). There was a significant main effect of age (*b* = −0.24, *SE* = 0.04, *p* < 0.001); the average stress levels were higher among younger women than older women. The main effect of number of children in the household (*b* = 0.04, *SE* = 0.04, *p* = 0.323) was not statistically significant, meaning that perceived stress levels did not differ by the number of children in the household. The interaction effect between number of children and age was not statistically significant (*b* = 0.04, *SE* = 0.04, *p* = 0.279). These results were consistent with those in Table 2 when children in the household was modeled as a dichotomous variable.

**Table 3:** Multiple regression models estimating the extent to which number of children in household is associated with perceived stress and anxiety.

	**Perceived stress**			**Anxiety**		
	***Est***	***SE***	***p***	***Est***	***SE***	***p***
Intercept	3.14	0.17	<0.001	1.35	0.16	<0.001
Number of children in household	0.04	0.04	0.323	−0.003	0.04	0.948
Age	−0.24	0.04	<0.001	−0.19	0.03	<0.001
Race (White)	0.10	0.14	0.505	0.17	0.14	0.207
Number of adults in household	0.06	0.04	0.102	0.05	0.04	0.165
Children × Age	0.04	0.04	0.279	0.08	0.04	0.035
*R^2^*	0.131			0.109		

*Perceived stress and anxiety were square root transformed. Number of children in household and age were centered. Age was divided by 10. Age, race, and number of adults in household were included as covariates. R^2^, proportion of variance explained*.

The main effect of number of children in household on anxiety was no longer statistically significant (*b* = −0.003, *SE* = 0.04, *p* = 0.948) when the number of children in the household was included as a continuous variable. The main effect of age (*b* = −0.19, *SE* = 0.03, *p* < 0.001), and interaction between number of children in household and age on anxiety remained statistically significant (*b* = 0.08, *SE* = 0.04, *p* = 0.035).

**Figure 1:**
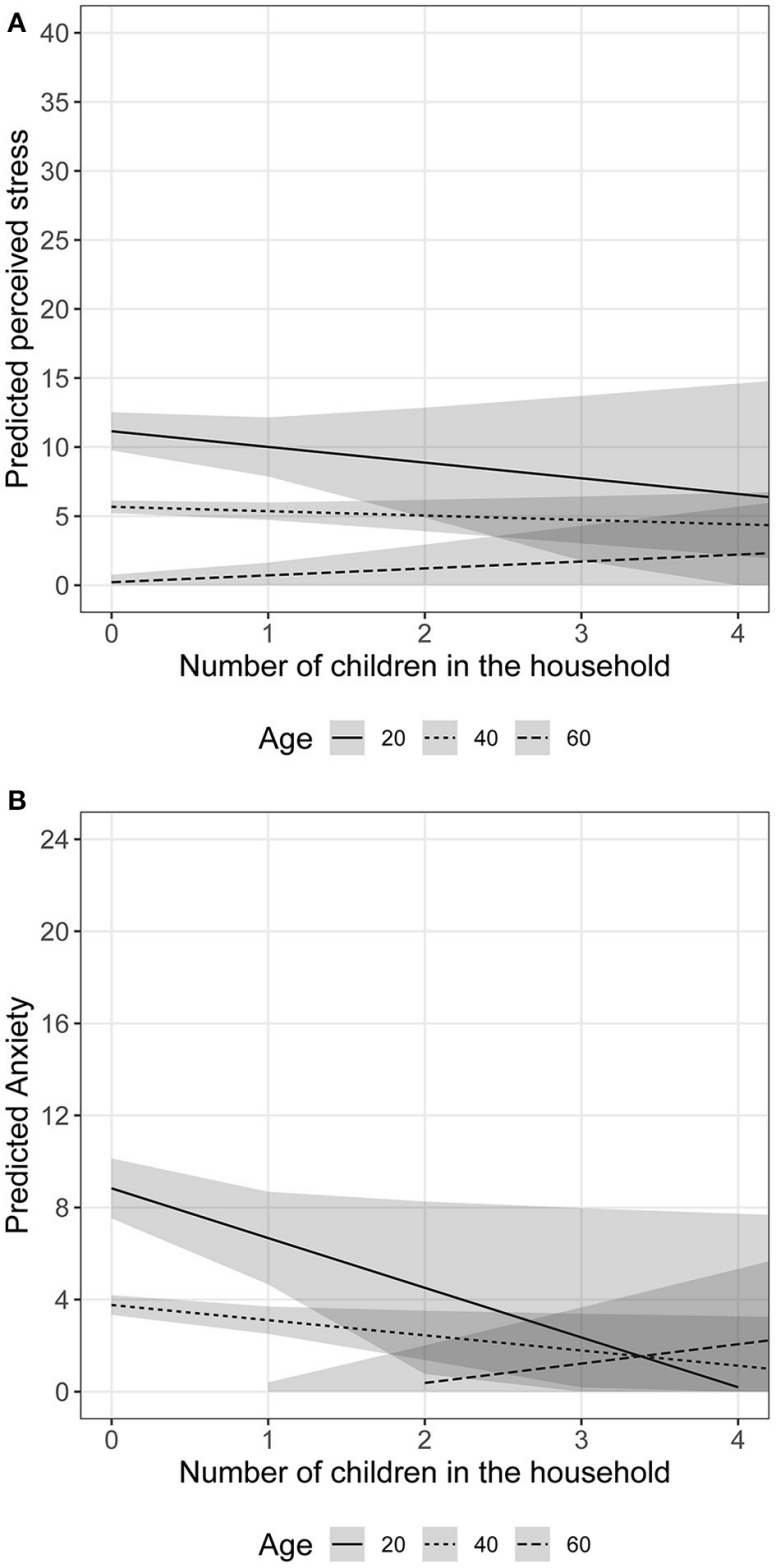
Associations between perceived stress/anxiety levels and number of children in the household among women of different age groups. Shaded area = 95% confidence interval. **(A)** Perceived stress and number of children in household among different age groups. **(B)** Anxiety and number of children in household among different age groups.

We illustrate the association between the number of children in the household and perceived stress/anxiety by age in Figure 1. The estimated coefficients in Table 3 are used to compute the predicted stress and anxiety scores, using different number of children and ages at 20, 40, and 60. As shown in the left panel (Figure 1A), stress levels decrease with age, reflecting the main effect of age on perceived stress. However, the slope illustrating the association between number of children and perceived stress is almost flat, reflecting that stress levels remain similar across women with different number of children. Figure 1B illustrates the interaction between number of children in household and age on anxiety. Among younger women (20- and 40-year-olds), anxiety levels decrease as the number of children in the household increases. However, anxiety levels increase with increasing number of children in the household among older women (60-year-olds).

### Discussion

Contrary to our hypothesis, perceived stress levels did not differ between women with and without children in the household. The average perceived stress levels were similar among women with different numbers of children in the household. In line with prior research, we found that older women had lower stress levels than younger women (43, 44), regardless of the number of children in the household (45, 46). Consistent with our hypothesis and with research elsewhere (47), average anxiety levels were higher among women living with children than those without children in the household. The effect of children in the household on anxiety levels was also different for women of different ages. It appears that younger women's anxiety levels decrease with increasing number of children in the household, whereas older women's anxiety levels increase as the number of children in the household increases.

However, it remains unclear whether the phenotypic relationship between children in the household and anxiety levels is due to non-random selection influences, causal effects, or both. We address this question in the study 2 using a co-twin control design.

## Study 2

In study 2, we aimed to replicate findings in our prior study (study 1) among female twin pairs. Furthermore, we examined whether the phenotypic associations between children in the household and mental health (i.e., stress and anxiety) is mediated by genetic and environmental factors shared within twin pairs. As twin pairs raised together share not only genetic influences (100% for identical twins and ~50% for fraternal twins), but also family and childhood environment; the use of twin pairs allowed a genetically informed design in which we can explore whether there are shared genetic and environmental factors that may be associated with both the presence of children in the household and mental health (i.e., stress and anxiety). As such, twin studies allow us to perform a more robust analysis than traditional correlational analysis among unrelated individuals by taking into account family-level selection factors (i.e., genetic and shared environmental influences).

### Methods

#### Participants

Study 2 utilized data from a sample of 529 female twin pairs [77.5% monozygotic (identical, MZ), 22.5% dizygotic (fraternal, DZ)] from the WSTR who participated in an online survey examining their feelings and daily activities during the COVID-19 pandemic. Details of the survey were described in study 1's Method section above. Zygosity was determined using five questions in the WSTR enrollment survey asking about childhood similarity. Compared to biological zygosity indicators, the survey items correctly classify zygosity with at least 95% accuracy (48).

#### Measures

Study 2 utilized the same measures as described in Study 1 above. As few participants had three or more children (5.1%), participants were categorized into three groups: no children (67.7%), one child (11.4%), and two or more children (20.9%).

#### Statistical Analysis

**Figure 2:**
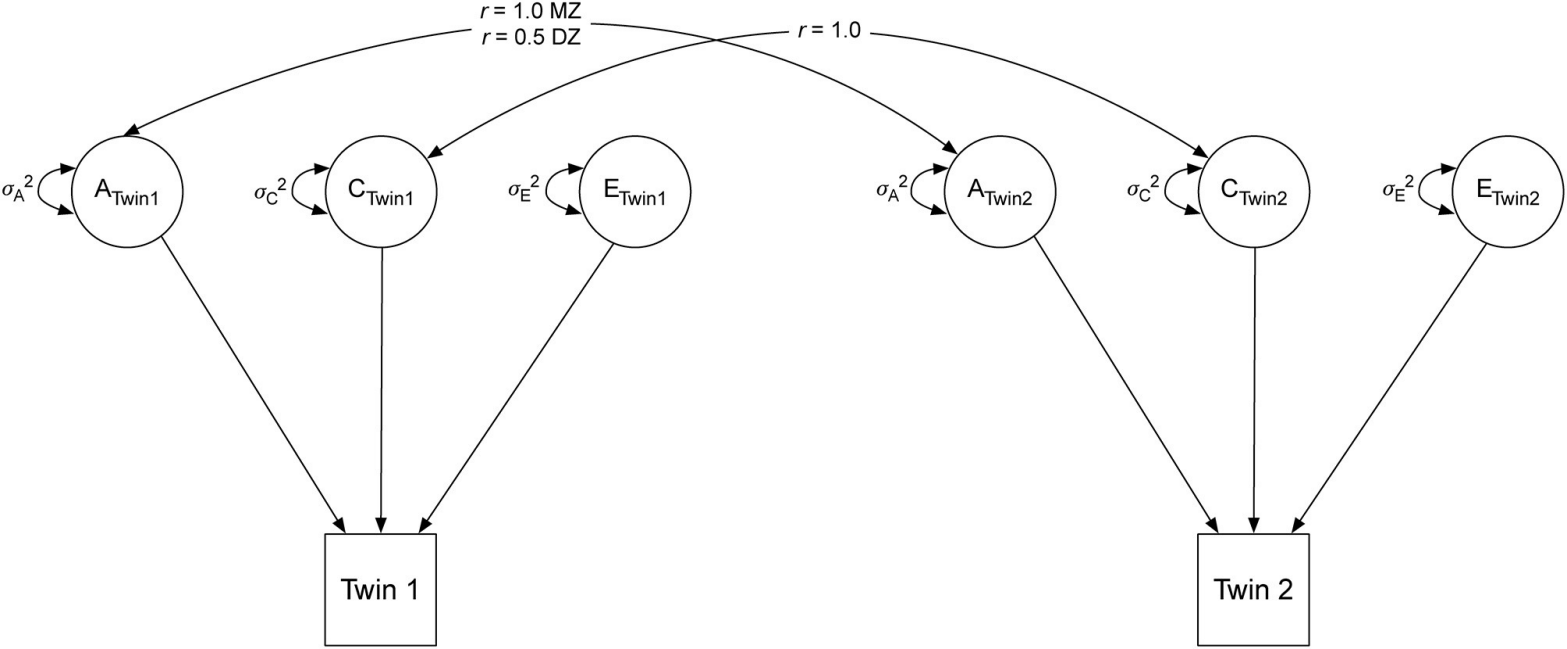
Univariate twin model. A, additive genetic component; C, shared environmental component; E, unique environmental component. MZ, monozygotic twins; DZ, dizygotic twins.

We first used the classical twin model to decompose the variances of number of children in the household, perceived stress, and anxiety into additive genetics (A), shared environmental (C), and non-shared environmental (E) components (Figure 2). The A variance components represent the additive effect of genes. As MZ twins share 100% of the additive genetic effects, the correlation between the A components (*r*_*A*_MZ) is 1.0; DZ twins share ~50% of the additive genetic effects, such that (*r*_*A*_DZ) is 0.5. The C variance components represent common, or shared, environmental experiences that make members of the same family more similar. By definition, the shared environment is perfectly correlated for MZ and DZ twins raised together, such that *r*_*C*_ = 1.0. The E variance components represent non-shared, or unique, environmental experiences; they do not correlate between twins and include measurement error (*r*_*E*_ = 0). Although we present the univariate variance decompositions of the three variables of interest, these were not the focus of the present study.

**Figure 3:**
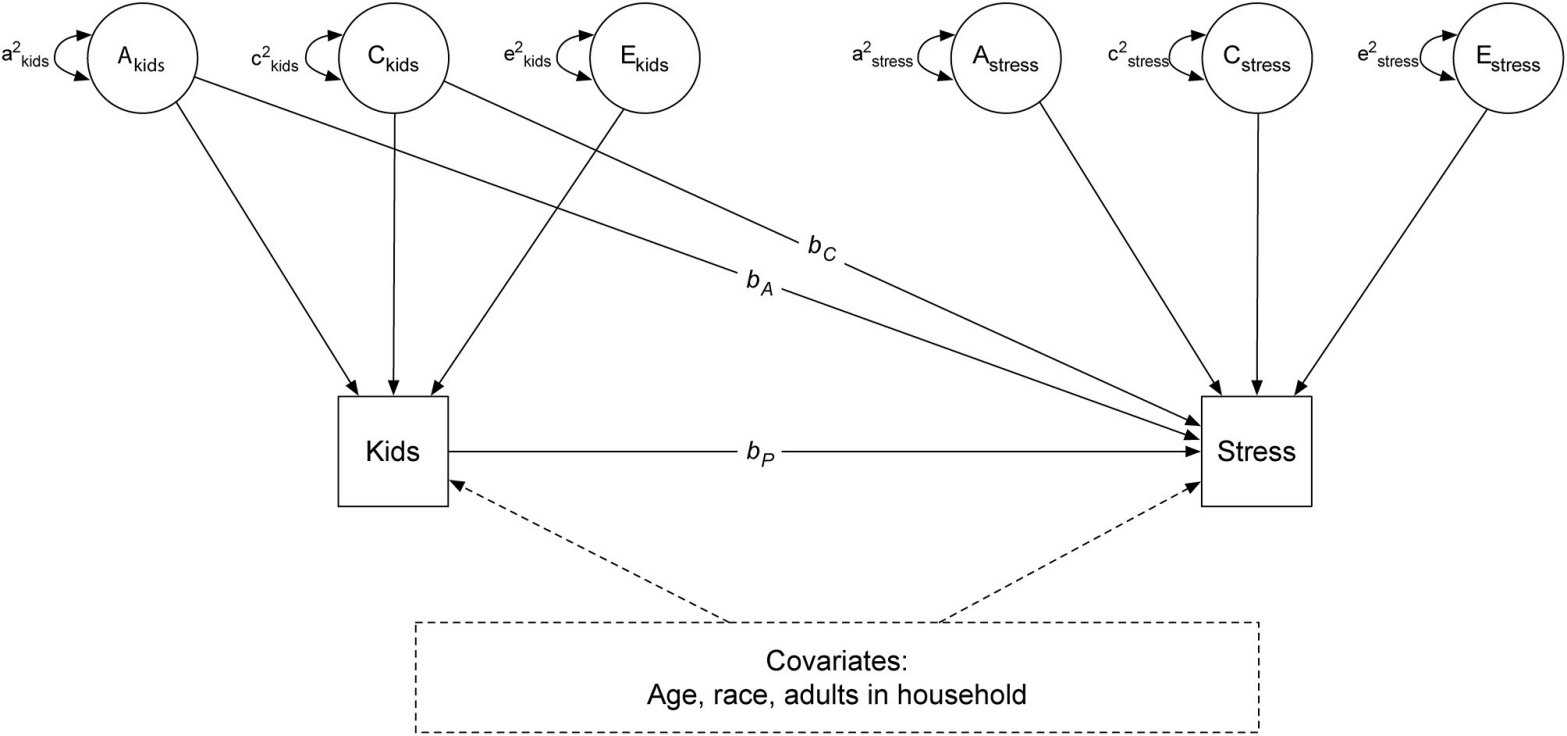
Bivariate twin model. A, additive genetic component; C, shared environmental component; E, unique environmental component.

We next used bivariate twin models to examine the extent to which number of children in the household is associated with mental health (i.e., perceived stress or anxiety). Detailed logic and methods are described in (49) and illustrated in Figure 3. The logic of the bivariate twin model is that if identical twins who differ in the number of children (i.e., one twin with more children and their cotwin with fewer children) also differ in mental health (i.e., one twin more stressed and their cotwin less stressed), the association between children and mental health cannot be genetically mediated as the twins share 100% of their DNA. On the other hand, if a pair of identical twins who differ in the number of children also differ in mental health, it is consistent with the hypothesis that the number of children causes worse mental health (i.e., more stress, more anxiety) at the level of the phenotype. As it is not possible to draw definitive inferences about causation without random assignment, we refer to such an association as “quasi-causal.”

As shown in Figure 3, stress is regressed on phenotypic number of children in the household (*b*_*p*_), the shared genetic (*b*_*A*_), and common environmental (*b*_*C*_) components of number of children in the household. In Model 1, *b*_A_ and *b*_*C*_ are set to zero; only the simple regression of stress on children in the household (*b*_*p*_) at the individual level is estimated. This model examines the association between children in the household and depression, without controlling for genetic or shared environmental confounds; it is referred to the phenotypic model.

In Model 2, estimates of *b*_*A*_ and *b*_*C*_, which controls for genetic and shared environmental confounds, are included in the estimation of the phenotypic effect. This model is referred to as a quasi-causal model (49). If the phenotypic association between children in the household and stress (*b*_*p*_) remains significantly different from zero after controlling for genetic and shared environmental confounds, it would be interpreted as a *quasi-causal* effect, meaning that stress levels differ within a pair of identical twins with different number of children in the household. If *b*_*p*_ is no longer statistically significant and reduced in magnitude after taking into account genetic and shared environmental confounds, a selection hypothesis is supported, reflecting no difference in stress levels between a pair of identical twins with different number of children. Finally, the model is estimated by including the set of covariates (age, gender, race, and number of adults in the household) previously described (Model 3). A similar set of models is performed examining the association between number of children in the household and anxiety.

Perceived stress and anxiety were positively skewed; they were square root transformed in all analyses. Age was divided by 10 to allow variables to be on similar scales. The shared genetic and environmental confounds (*b*_*A*_ and *b*_*C*_) were initially estimated with large standard errors, suggesting that the parameters were not estimated with precision, indicating insufficient power to distinguish between shared genetic and shared environmental influences. We therefore constrained *b*_*A*_ and *b*_*C*_ to equality, meaning that only between-family confounds are estimated (i.e., *b*_*between*_) in all subsequent models.

Descriptive statistics were performed in the statistical program R 4.0.2 (42). All latent variable path analyses were conducted using the computer program Mplus v. 8.1 (50). The alpha level for testing hypotheses was set to 0.05. Twin-based regression models are generally saturated; the only source of reduced fit involves incidental issues such as differences between twins arbitrarily assigned as Twin 1 and Twin 2 within pairs. All reported models fit the data closely using standard “goodness of fit” tests.

### Results

#### Descriptive Statistics

Table 4 shows the descriptive statistics of select demographic characteristics in the current female twins sample. More than half of the participants reported not living with any children (67.7%), with smaller proportions having one (11.4%) or two or more (20.9%) children in the household. Cronbach's alpha for the PSS (0.90, 95% CI: 0.89, 0.91) and the anxiety scale (0.84, 95% CI: 0.82, 0.85) suggested good to excellent reliability.

**Table 4:** Descriptive statistics of select demographic characteristics, number of children and adults in household, perceived stress, and anxiety in the Washington State Twin Registry (WSTR) same-sex twins sample in study 2.

	***n* = 1,058**
Age	49.5 (*15.5*)
White	1,012 (95.7%)
**Zygosity**	
MZ	820 (77.5%)
DZ	238 (22.5%)
Number of adults in household	2 [1–10]
**Number of children in household**	
0	711 (67.7%)
1	120 (11.4%)
2+	219 (20.9%)
**Perceived stress**	
0 children	11.1 (*6.7*)
1 child	13.0 (*8.1*)
2+ children	13.7 (7.0)
**Anxiety**	
0 children	3.2 (*3.3*)
1 child	3.8 (*3.9*)
2+ children	3.7 (*3.6*)

*Means (standard deviations) are presented for continuous variables. Frequencies (proportions) are presented for categorical variables. Median [range] is presented for the number of adults in household. MZ, monozygotic twins; DZ, dizygotic twins*.

#### Effect of Number of Children on Perceived Stress

Results of the univariate twin models were reported in the Supplementary Materials. Results of the bivariate twin models examining the effect of the number of children on perceived stress were presented in Table 5. In Model 1, there was a significant phenotypic association between the number of children and perceived stress (*b*_*p*_ = 0.21, *SE* = 0.04, *p* < 0.001). Twins with more children in the household were more likely to have higher stress levels than those with fewer children in the household; an increase of one child was associated with a less than one-unit increase^2^ in perceived stress. In Model 2, this relationship was no longer statistically significant (*b*_*p*_ = 0.05, *SE* = 0.18, *p* = 0.767) after taking into account between-family confounds (*b*_*between*_ = 0.19, *SE* = 0.23, *p* = 0.423). Results remained similar after controlling for age, race, and number of adults in the household in Model 3.

**Table 5:** Unstandardized parameter estimates for phenotypic and biometric models estimating the effects of children in household on perceived stress levels.

	**Model 1**		**Model 2**		**Model 3**	
	**Phenotypic model**		**Quasi-causal model**		**Quasi-causal model**	
	**Est (*SE*)**	***P***	**Est (*SE*)**	***p***	**Est (*SE*)**	***p***
*b_*between*_*			0.19 (*0.23*)	0.423	0.09 (*0.23*)	0.680
*b_*p*_*	0.21 (*0.04*)	<0.001	0.05 (*0.18*)	0.767	0.07 (*0.16*)	0.656
**Covariates**						
Age					−0.22 (*0.06*)	0.001
White					−0.14 (*0.18*)	0.437
Adults in household					0.363 (*0.33*)	0.264
RMSEA	0.040 [0.022, 0.058]	0.041 [0.023, 0.059]	0.036 [0.025, 0.047]			
CFI	0.966	0.966	0.950			
TLI	0.979	0.978	0.949			

*b_A_, amount of variance in fear of perceived stress due to additive genetic influences; b_P_, phenotypic association between children in household and perceived stress; SE, standard error; RMSEA, Root mean square error of approximation; CFI, comparative fit index; TLI, Tucker-Lewis index. Phenotypic model does not include controls for between-pair confounds, whereas quasi-causal models include controls for between-pair confounds. Age is divided by 10*.

**Figure 4:**
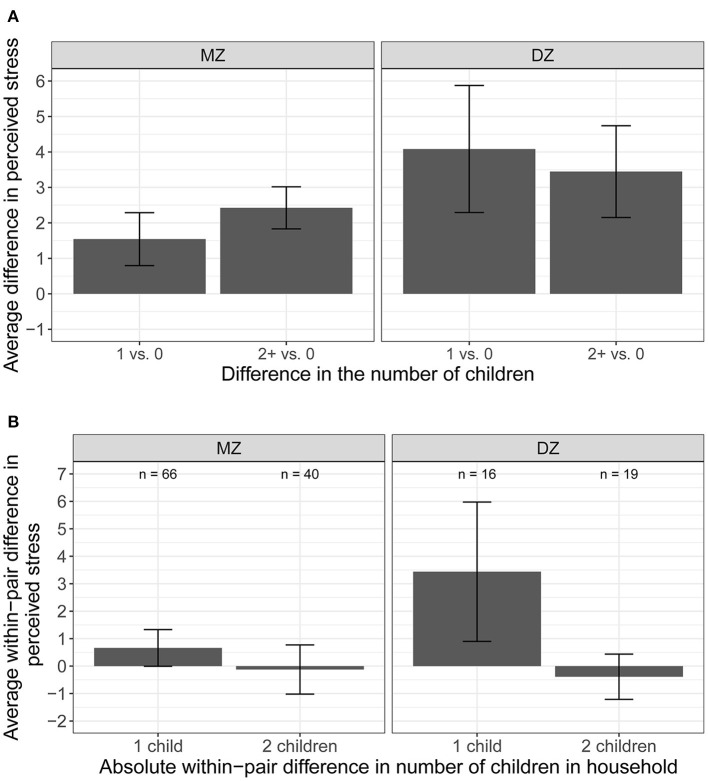
Average difference and within-pair difference in perceived stress levels and children. **(A)** Average difference in perceived stress between participants with no children and one/two or more children in the household. **(B)** Average difference in perceived stress between member of the pair with children and the co-twin without children in the household.

We illustrate the differences between the significant phenotypic association and the non-significant quasi-causal effect in Figure 4. In Figure 4A, we plotted the average difference in perceived stress levels between participants with children (one, and two or more) and those with no children in the household. Compared to individuals without children, perceived stress levels were higher in those with children, illustrating the population-level association between number of children and perceived stress. Figure 4B illustrates the within-pair difference in perceived stress between twin pairs discordant in the number of children (i.e., one member of the pair has children, whereas the co-twin does not have children in the household; pairs with the same number of children were not included), stratified by the magnitude of the difference (one, or two or more children). There is no visible effect of number of children within MZ pairs, meaning that stress levels are similar (difference in perceived stress level is close to zero) within a pair of MZ twins with different number of children in the household (left panel). This reflects the non-significant quasi-causal effect of number of children on perceived stress reported in Table 3, suggesting that the phenotypic association between number of children and perceived stress is mediated by between-family confounds. Of note, the number of DZ twin pairs discordant in number of children was very small (*n* = 16 for one-child difference, and *n* = 19 for two or more children difference). The large standard errors reflected large variation in the estimated within-pair difference among DZ twins, suggesting that the within-pair difference in stress levels may not be estimated with precision.

#### Anxiety on Number of Children

As shown Table 6, there was a significant phenotypic association between the number of children and anxiety (*b*_*p*_ = 0.08, *SE* = 0.03, *p* < 0.016; Model 1). Twins with more children in the household were more likely to have higher anxiety levels than those with fewer children in the household. The effect was small; an increase of one child was associated with less than a one-tenth unit increase in anxiety. This relationship was no longer statistically significant (*b*_*p*_ = −0.18, *SE* = 0.16, *p* = 0.278) after taking into account between-family confounds (*b*_*between*_ = 0.32, *SE* = 0.21, *p* = 0.133) in Model 2. Model 3 showed consistent results after including age, race, and number of adults in the household as covariates.

**Table 6:** Unstandardized parameter estimates for phenotypic and biometric models estimating the effects of children in household on anxiety levels.

	**Model 1**		**Model 2**		**Model 3**	
	**Phenotypic model**		**Quasi-causal model**		**Quasi-causal model**	
	**Est (*SE*)**	***P***	**Est (*SE*)**	***p***	**Est (*SE*)**	***p***
*b_*between*_*			0.32 (*0.21*)	0.133	−0.001 (*0.21*)	0.996
*b_*p*_*	0.08 (*0.03*)	0.016	−0.18 (*0.16*)	0.278	−0.10 (*0.14*)	0.472
**Covariates**						
Age					−0.24 (*0.06*)	<0.001
White					0.10 (*0.14*)	0.468
Adults in household					0.58 (*0.24*)	0.015
RMSEA	0.043 [0.026, 0.061]	0.043 [0.026, 0.061]	0.037 [0.026, 0.047]			
CFI	0.957	0.959	0.943			
TLI	0.973	0.973	0.942			

*b_A_, amount of variance in anxiety attributable to additive genetic influences; b_P_, phenotypic association between children in household and anxiety; SE, standard error; RMSEA, Root mean square error of approximation; CFI, comparative fit index; TLI, Tucker-Lewis index. Phenotypic model does not include controls for between-pair confounds, whereas quasi-causal models include controls for between-pair confounds. Age is divided by 10*.

**Figure 5:**
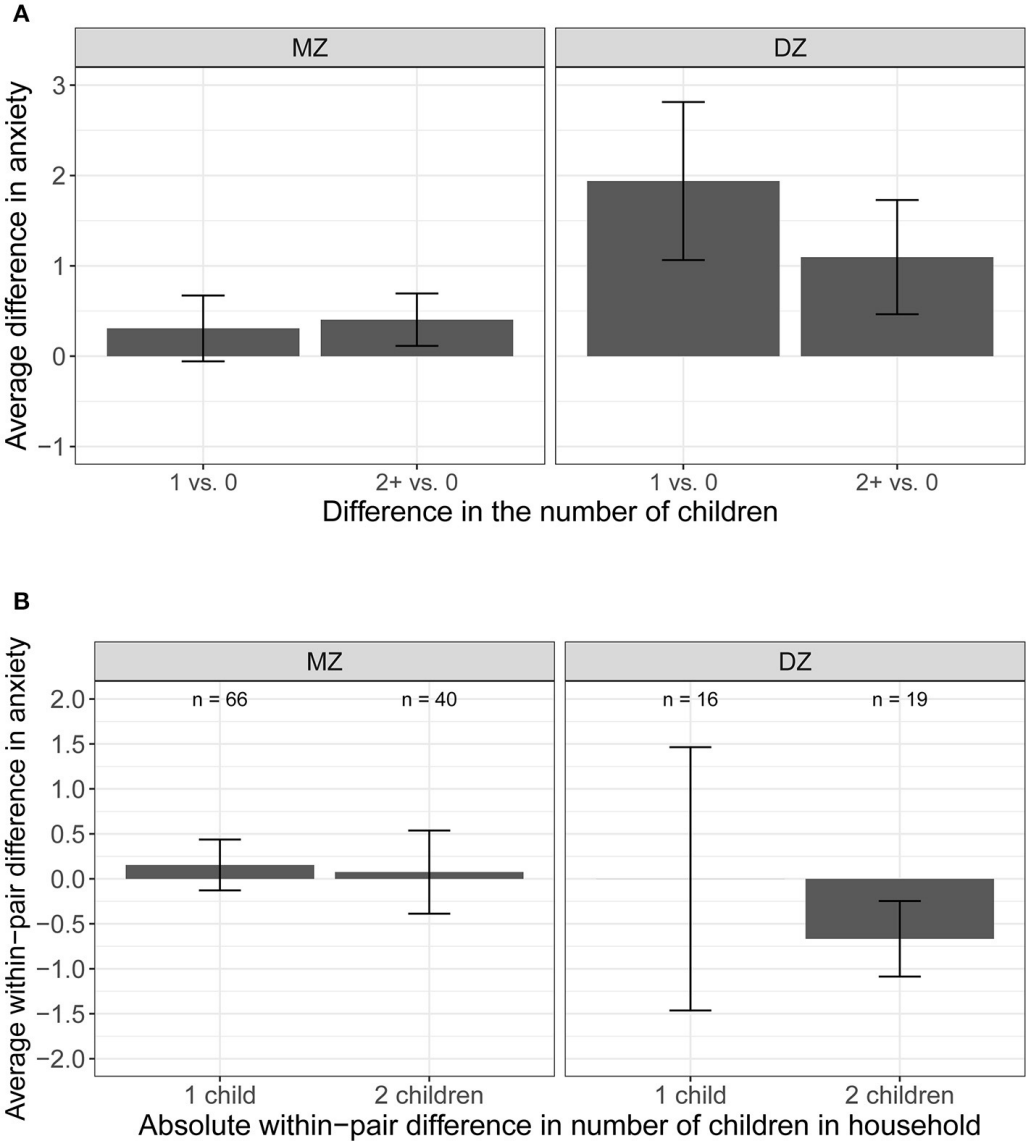
Average difference and within-pair difference in anxiety levels and children. **(A)** Average difference in anxiety between participants with no children and one/two or more children in the household. **(B)** Average difference and within-pair difference in anxiety levels between kids.

These results are illustrated in Figure 5. We observed a small difference in the average anxiety levels between participants with children (one, and two or more) and those with no children in the household (Figure 5A). The average anxiety levels were slightly higher for participants with children vs. those with no children, illustrating the small phenotypic association between the number of children and anxiety. The within-pair difference in anxiety between the member of the pair with children and the member of the pair with no children in the household by the magnitude of the difference (one, or two or more children; pairs with the same number of children were not included) was shown in Figure 5B. There is no visible effect of number of children on anxiety levels within pairs—the average within-pair difference in anxiety level is close to zero.

### Discussion

The current study showed that the presence of children in the household is associated with higher levels of stress and anxiety, partially replicating our findings in the study 1. However, once between-family influences are taken into account, the associations are attenuated and no longer significant, suggesting that genetic and shared environmental factors confounded the effect of children in the household on stress and anxiety. Findings from study 2 suggest that the observed association between number of children in the household and stress/anxiety levels is mediated by between-family influences shared within twin pairs.

## Overall Discussion

This paper adds to a growing body of literature showing the negative impacts of the COVID-19 pandemic on mental health in parents, specifically mothers and/or women living with children under 18. In study 1, we showed that women living with children were more anxious, but not more stressed, than those living without children. Our findings are consistent with a study of Italian women which also found a higher level of anxiety in mothers compared to women without children (28). Another recent study also reported a small effect of number of children in the household on depression during the first few months of the COVID-19 pandemic; self-reported depression levels were slightly higher among those living with more children than those with fewer children in the household (33). In our sample, levels of stress decreased with age, which has also been reported elsewhere (43, 44). Taken together, it appears that having children in the household may be mentally taxing for women during the pandemic.

In study 2, we showed that the association between number of children in the household and stress/anxiety levels was confounded by between-family factors shared within twin pairs. Among twin pairs living with different numbers of children, there was no observable differences in stress and anxiety levels between the member of the pair living with more children and their co-twin living with fewer children. Our findings suggest that the population level association observed between number of children in the household and stress/anxiety levels is mediated by early environmental factors shared within twin pairs.

Consistent in our two studies, we found that older women were less stressed and anxious than younger women, though the effect was relatively small. This finding is consistent with other studies that showed older adults were less negatively affected than younger adults by the pandemic (51). When we examined the effect of the number of children in the household on women's anxiety levels, we found that older women living with more children were more anxious than those living with fewer children, whereas younger women living with more children were less anxious than those living with fewer children. It is possible that the trend of older adults being more mentally resilient during this time period may be limited to those who live with no, or few, children, as they are better able to follow social distancing guidelines and limit social contact. On the other hand, older adults who live with more children may experience elevated anxiety due to their limited ability to distance themselves from other people, and/or the extra caregiving burden during this time. Younger women with more children may be used to engaging with family and friends via social media or electronic communication or connecting with other mothers in online groups for support, resulting in lower levels of anxiety despite the reduced physical social contact.

As society navigates toward a “new normal” with the COVID-19 pandemic, it is important to recognize that certain groups of individuals may have experienced higher levels of stress and anxiety. Our current findings add to the existing literature that the COVID-19 pandemic has a negative effect on mental health for women (52–54), especially those living with children. Pandemic-related stress includes income loss, lack of nutritious food choices, mental health challenges, limited access to health services, and increased risk of violence (54). Left unchecked, these stressors may be associated with increased physical and mental health issues over time. Moving forward, resources should be invested in helping women as society slowly returns to normal. For example, COVID-19 relief and/or related financial aid programs should adjust application requirements to ensure that women, especially women living with children, are able to access aid independently. Community support groups should be available to help women resume contact with family and friends, offer support and/or access to childcare services, easy access to psychological support, and/or access to safe spaces for those in need.

### Strengths and Limitations

One of the major strengths of this paper was the timeliness of the survey. Participants in sample 1 responded to our survey less than a month after the World Health Organization declared COVID-19 a pandemic on March 11. Online surveys were administered to participants in sample 2 within 2 months of COVID-19 being declared a pandemic. As such, we were able to assess the extent to which children in the household were associated with stress and anxiety levels among adult women as they coped with the impact of the COVID-19 pandemic and social mitigation strategies implemented by local and state governments. Second, the use of female twin pairs in study 2 allowed us to explore whether the association between number of children in the household and women's mental health is due to non-random selection, causal mechanisms, or both. By utilizing a co-twin control design, we were able to take into account between-family confounds (i.e., genetic and shared environmental factors) that are otherwise uncontrolled in traditional correlation analyses. In the current study, we showed that the phenotypic association between number of children and women's anxiety levels was confounded by non-random confounds shared within twin pairs.

A number of limitations in the present study should be noted. The relationship between the children in the household and the participants was not assessed in the adult twins samples (i.e., sample 1 in study 1 and all participants in study 2). As only the presence of children 0–17 in the household was assessed, it is possible that some respondents live with their own children under the age of 18, but it is also possible that some participants live with other relatives under the age of 18 (e.g., nieces, nephews, and/or grandchildren). More than 2.5 million grandparents are raising their grandchildren in the US (55). Job loss due to COVID-19 has forced many Americans to move back home with their parents, which may include their older children over the age of 18. These temporary arrangements, combined with the uncertainty of the COVID-19 pandemic, may be associated with stress and anxiety as well. We were unable to investigate whether the ages of the children have an impact on women's stress and anxiety as children's ages were not assessed in our studies. Prior research has shown that parents of minor children experience more distress than parents of adult children or childless individuals (19, 56). Future research should consider examining whether stress and anxiety levels of mothers decrease as schools reopen and social restriction measures are relaxed in the coming months. Relatedly, we were unable to assess whether the association between children in the household and mental health is moderated by relationship with the children, marital status and/or social support as these questions were not included in the survey. A study of perceived stress in mothers during COVID-19 found that the cumulative number of COVID-19 related stressors, such as changes in one's relationship with their partner, changes in interactions with child(ren), and changes in child(ren)'s academics, was positively associated with perceived stress (32). While depression levels within the first few months of the COVID-19 pandemic were lower among married and/or cohabiting adults than those not married or living with a partner (33), the current study did not examine differences in depressive feelings as our sample self-reported few depressive symptoms. Marital status was found to be protective against mental disorders among parents (57); support from spouse, family, and friends has also been shown to be associated with decreases in anxiety and stress among mothers (18). Additional research is needed to better understand the complex relation between children and mental health of adults in the same household.

In addition, it is possible that perceived stress in general does not necessarily reflect parental stress. Parents may experience differing levels of stress about COVID-19 and stress directly related to parenting. We recognize that it is possible that individuals' stress and anxiety may differ by geographical locations. Although we did not ask about geographical location in this study, most of the members enrolled in the WSTR live in Washington State, primarily in the Puget Sound area. Given that the Puget Sound was the initial epicenter of COVID-19 in the United States (58, 59), future research should examine whether stress and anxiety levels differed by geographic location, give the differences in COVID-19 cases, vaccination rates, and availability of healthcare. Finally, due to the cross-sectional nature of the current data, we are unable to determine whether the number of children in the household leads to elevated anxiety levels in this paper. However, as the WSTR continues to follow these respondents over time during the COVID-19 pandemic, it may be possible to investigate whether the changes in stress and anxiety levels are associated with changes in the number of children in the household and/or other additional influences.

## Conclusion

This paper examined the extent to which the number of children in the household was associated with levels of stress and anxiety among women within the first few months of the COVID-19 pandemic. We found that perceived stress levels were similar among women living with and without children in the household, whereas anxiety levels were, on average, higher among women living with children than those living without children. We further showed that the association between number of children in the household and stress/anxiety levels was confounded by between-family factors shared within twin-pairs, suggesting that this relationship is mediated by the environment shared between members of the twin pair. Of note, the association between children in the household and anxiety levels differed among women of different ages; older women were more anxious with more children in the household, whereas younger women were less anxious with more children in the household. Findings in the current study highlight the need to provide supporting resources to women living with children in the household during the COVID-19 pandemic. Childcare and/or education resources that can help alleviate some of the burden placed on women, especially older women, would potentially be helpful in reducing the amount of anxiety they may be experiencing during this difficult time.

## Data Availability Statement

The datasets presented in this article are not readily available because data is available from the Washington State Twin Registry after application and approval for use. Requests to access the datasets should be directed to ws.twinregistry@wsu.edu.

## Ethics Statement

The studies involving human participants were reviewed and approved by Washington State University Institutional Review Board. Written informed consent for participation was not required for this study in accordance with the national legislation and the institutional requirements.

## Author Contributions

AA, ST, ES, and GD contributed to the design and implementation of the research, to the analysis of the results, and to the writing of the manuscript. All authors contributed to the article and approved the submitted version.

## Conflict of Interest

The authors declare that the research was conducted in the absence of any commercial or financial relationships that could be construed as a potential conflict of interest.

## Publisher's Note

All claims expressed in this article are solely those of the authors and do not necessarily represent those of their affiliated organizations, or those of the publisher, the editors and the reviewers. Any product that may be evaluated in this article, or claim that may be made by its manufacturer, is not guaranteed or endorsed by the publisher.

## Footnotes

^1^Only singleton respondents are included in study 1 of this paper, as the use of twin pairs violates the assumption of independence for the statistical analyses. Furthermore, data from twin pairs are analyzed using co-twin control design in the study 2 of the current paper.

^2^The increase is less than one-unit increase as perceived stress was square root transformed in the analyses.
