# Supplementary material for: Differences in Stress and Anxiety Among Women With and Without Children in the Household During the Early Months of the COVID-19 Pandemic
Source: Front Public Health. 2021 Sep 1;9:688462. doi: 10.3389/fpubh.2021.688462 (PMC8440851; doi:10.3389/fpubh.2021.688462)
Supplement: Supplementary file 1 [file Table_1.DOCX]

*Supplementary table 1*. Results of echocardiography before (T_0_) and at the end of the cycle (T_1_) with AAS, and after the recovery period (T_2_). Mixed models were used for trend analysis. The mean is shown with 95%-confidence interval (CI). ^†^P=.01 to .05, ^‡^P<0.01.

|  | **T_0_ (n=31) x̄ [CI]** | **T_1_ (n=31) x̄ [CI]** | **T_2_ (n=25) x̄ [CI]** |
| --- | --- | --- | --- |
| **Basic parameters** |  |  |  |
| Heart rate  (/min) | 59.3 (n=31) [56.2 to 62.4] | 69.3^‡^ (n=31) [66.2 to 72.4] | 61.6 (n=25) [0.3 to 1.6] |
| Systolic blood pressure  (mmHg) | 130 (n=31) [126 to 134] | 136^†^ (n=31) [132 to 140] | 127 (n=25) [122 to 132] |
| Diastolic blood pressure (mmHg) | 81 (n=31) [77 to 85] | 86^†^ (n=31) [82 to 89] | 79 (n=25) [75 to 83] |
| Body surface area  (BSA, m^2^) | 2.15 (n=31)  [2.09 to 2.21] | 2.21^†^ (n=31) [2.25 to 2.26] | 2.11 (n=25) [2.05 to 2.18] |
|  | | | |
| **Left ventricle** |  |  |  |
| Left ventricular end-diastolic dimension (LVEDd, mm) | 52.8 (n=31) [51.0 to 54.7] | 53.6 (n=30) [51.8 to 55.5] | 53.4 (n=25) [51.4 to 55.5] |
| Left ventricular end-systolic dimension (LVEDs, mm) | 35.0 (n=31) [33.4 to 36.6] | 34.9 (n=30) [33.3 to 36.5] | 36.6 (n=25) [34.8 to 38.4] |
| Left ventricular end-diastolic  volume 3D (3D LVEDV, ml) | 184.1 (n=26) [172.5 to 195.7] | 194.5^†^ (n=28) [183.0 to 205.9] | 167.3^‡^ (n=18) [155.4 to 179.1] |
| Left ventricular end-systolic  volume 3D (3D LVESV, ml) | 74.4 (n=25) [67.2 to 81.6] | 85.9^‡^ (n=28) [78.8 to 93.0] | 66.4^‡^ (n=17) [59.1 to 73.8] |
| Intraventricular end-diastolic  septal thickness (IVSd, mm) | 8.21 (n=31) [7.81 to 8.62] | 9.08^‡^ (n=31) [8.68 to 9.49] | 8.51 (n=25) [8.07 to 8.95] |
| Left ventricular end-diastolic posterior wall thickness (LVPWD, mm) | 8.18 (n=31) [7.75 to 8.60] | 9.36^‡^ (n=31) [8.94 to 9.78] | 8.45 (n=25) [8.00 to 8.91] |
| Left ventricular mass  (LV mass, g) | 161.5 (n=31) [146.4 to 176.5] | 189.8^‡^ (n=30) [174.6 to 205.0] | 165.8 (n=25) [149.7 to 181.9] |
| Left ventricular ejection fraction 3D (3D LVEF, %) | 61.1 (n=26) [58.8 to 63.4] | 56.2^‡^ (n=28) [54.0 to 58.5] | 60.8 (n=24) [58.4 to 63.2] |
| Global left ventricular strain  (LV strain global, %) | -18.9 (n=25) [-20.2 to -18.0] | -18.0 (n=22) [-19.1 to -16.9] | -19.1 (n=21) [-20.2 to -18.0] |
| Myocardial performance index  (MPI, Tei-index) | 0.34 (n=27) [0.29 to 0.40] | 0.42 (n=22) [0.35 to 0.48] | 0.36 (n=21) [0.30 to 0.42] |
|  |  |  |  |
| **Diastolic function** |  |  |  |
| Mitral valve E wave  (E, cm/s) | 73.4 (n=30) [67.7 to 79.0] | 69.2 (n=29) [63.4 to 74.9] | 72.0 (n=25) [65.9 to 78.1] |
| Mitral valve E wave duration time (E-DT, ms) | 209 (n=29) [193 to 225] | 204 (n=29) [189 to 221] | 215 (n=25) [198 to 233] |
| Mitral valve A wave  (A, cm/s) | 44.4 (n=30) [39.3 to 49.4] | 54.5^‡^ (n=29) [48.3 to 58.7] | 43.9 (n=25) [38.4 to 49.4] |
| Mitral valve A wave duration time (A-DT, ms) | 151 (n=30) [142 to 161] | 144 (n=28) [134 to 153] | 151 (n=25) [141 to 161] |
| E/A-ratio | 1.80 (n=31) [1.59 to 2.01] | 1.35^‡^ (n=22) [1.14 to 1.56] | 1.78 (n=25) [1.56 to 2.01] |
| Lateral e’ wave  (e’ lat, cm/s) | 15.1 (n=31) [13.7 to 16.5] | 13.3^†^ (n=31) [11.9 to 14.6] | 14.2 (n=25) [12.7 to 15.7] |
| Septal e’ wave  (e’ sept, cm/s) | 10.6 (n=31) [9.8 to 11.5] | 9.6 (n=30) [8.8 to 10.5] | 10.2 (n=25) [9.2 to 11.1] |
| Lateral E/e’-ratio  (E/e’ lat) | 4.9 (n=30) [4.2 to 5.7] | 5.5 (n=29) [4.8 to 6.3] | 5.9 (n=25) [5.1 to 6.7] |
| Septal E/e’-ratio  (E/e’ sept) | 7.2 (n=30) [6.5 to 7.9] | 7.6 (n=28) [6.9 to 8.3] | 7.2 (n=25) [6.5 to 8.0] |
| Pulmonary vein Arev  (PV Arev, cm/s) | 33.8 (n=25) [26.8 to 40.9] | 30.5 (n=28) [23.8 to 37.1] | 32.0 (n=23) [24.6 to 39.4] |
| Pulmonary vein Arev duration time (PV Arev DT, ms) | 107 (n=26) [97 to 117] | 114 (n=28) [104 to 123] | 116 (n=23) [105 to 127] |
| Pulmonary vein D  (PVD, cm/s) | 54.4 (n=26) [50.0 to 58.8] | 53.6 (n=30) [49.5 to 57.8] | 51.2 (n=23) [46.4 to 55.9] |
| Pulmonary vein S  (PVS, cm/s) | 49.0 (n=26) [44.7 to 53.3] | 51.8 (n=30) [47.8 to 55.9] | 45.8 (n=23) [41.2 to 50.4] |
| Left atrial volume 3D  (3D LAvol, ml) | 62.1 (n=25) [56.6 to 67.5] | 71.2^‡^ (n=27) [65.9 to 76.5] | 63.5 (n=23) [57.8 to 69.3] |
|  | | | |
| **Right ventricle** |  |  |  |
| Tricuspid annular plane systolic excursion (RV TAPSE, mm) | 24.6 (n=24) [23.2 to 26.1] | 23.2 (n=27) [21.8 to 24.6] | 24.3 (n=24) [22.9 to 25.7] |
| Right ventricular tissue Doppler imaging (RV TDI, cm/s) | 13.4 (n=22) [12.6 to 14.2] | 13.1 (n=25) [12.4 to 13.9] | 13.4 (n=25) [12.6 to 14.1] |
| Right ventricular annulus diastolic (RV annulus, mm) | 38.1 (n=30) [36.2 to 40.0] | 38.9 (n=29) [37.0 to 40.8] | 38.4 (n=24) [36.3 to 40.4] |
| Right ventricular fractional area change (RVFAC, %) | 49.2 (n=30) [45.6 to 52.9] | 48.1 (n=30) [44.5 to 51.8] | 50.7 (n=25) [46.7 to 54.8] |
| Pulmonary artery acceleration slope (PA acc slope, cm/s^2^) | 671 (n=25) [534 to 808] | 772 (n=27) [638 to 906] | 674 (n=24) [532 to 817] |
| Pulmonary artery acceleration time (PAAT, ms) | 160 (n=25) [146 to 174] | 143 (n=27) [129 to 157] | 158 (n=24) [143 to 173] |
| Right ventricular outflow tract  (RVOT, mm) | 34.0 (n=30) [32.1 to 35.9] | 34.8 (n=28) [32.9 to 36.8] | 33.6 (n=24) [31.5 to 35.6] |
| Right ventriculo-arterial coupling (RVAC) | 49.3 (n=27) [45.4 to 53.1] | 47.9 (n=29) [44.2 to 51.6] | 50.4 (n=23) [46.2 to 54.6] |
